# P-604. Viral Inhibition Efficacy from Lambda Carrageenan Demonstrated Against Multiple Respiratory Viral Pathogens Including Avian Influenza H5N1

**DOI:** 10.1093/ofid/ofaf695.817

**Published:** 2026-01-11

**Authors:** James W Arbogast, Cory Chiossone, Tanya Kapes, Susan Nicholson

**Affiliations:** JW Arbogast Advanced Science Consulting LLC, Akron, OH; Microbac Laboratories, Inc., Sterling, Virginia; Microbac Laboratories, Inc., Sterling, Virginia; Lanvira LLC, New York, New York

## Abstract

**Background:**

Respiratory viral infections remain a significant global health challenge, particularly with the emergence and re-emergence of highly pathogenic strains such as avian influenza H5N1 (Influenza A virus subtype H5N1). The search for broad-spectrum antiviral agents and prophylactic products has intensified in recent years. Lambda (λ) carrageenan, a sulfated polysaccharide derived from red seaweed, has antiviral properties, and is the most potent antiviral carrageenan type. This study investigates the efficacy of λ-carrageenan in inhibiting a range of respiratory viral pathogens through multiple in vitro assays, with a specific focus on its activity against avian influenza H5N1.

**Table 1.**
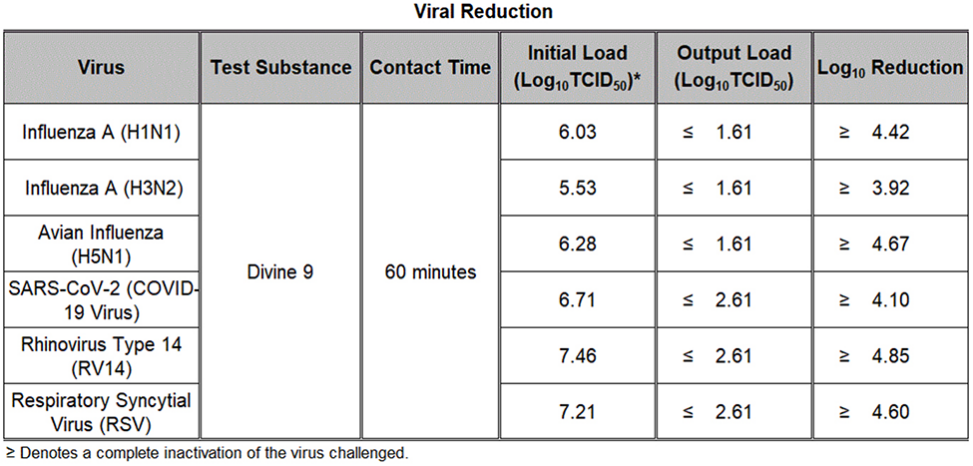
Efficacy Results Summary

**Table 2:**
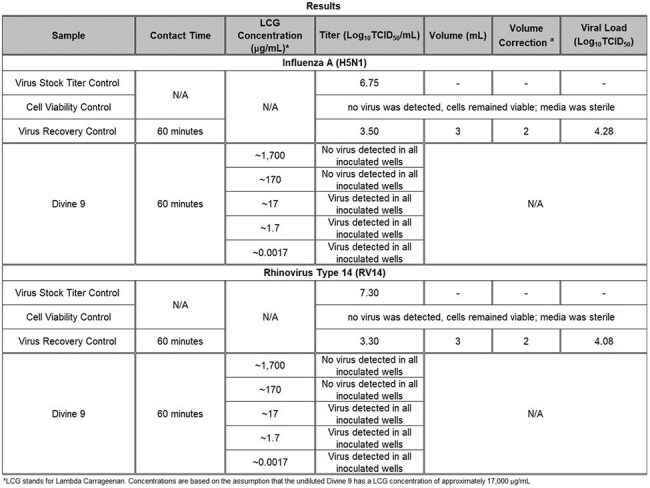
Titration Study Test Results

**Methods:**

A λ-carrageenan based product was tested as delivered from a commercial personal lubricant sample (Divine 9) using ASTM E1052 (“Standard Practice to Assess the Activity of Microbicides against Viruses in Suspension”). The test article was analyzed either quantitatively or qualitatively following the established protocols with laboratory techniques appropriate for each virus tested.

**Table 3:**
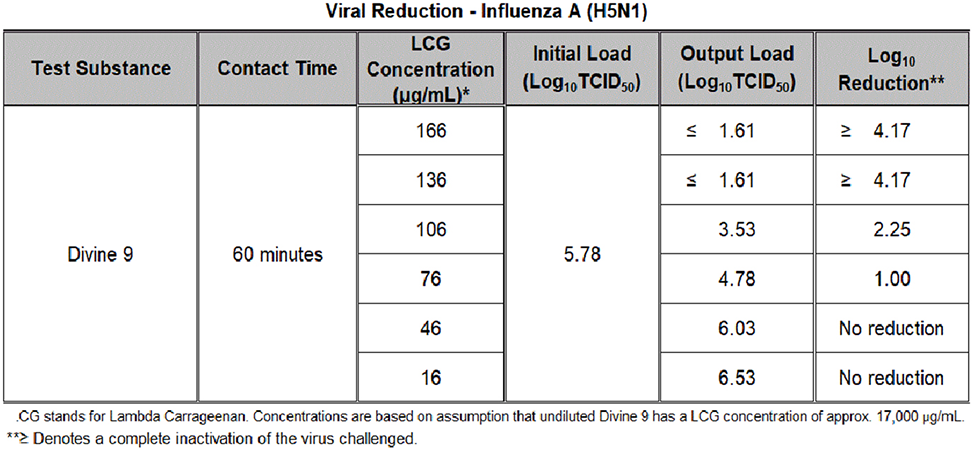
Inhibitory Concentration Test Results for Avian Influenza H5N1

**Table 4:**
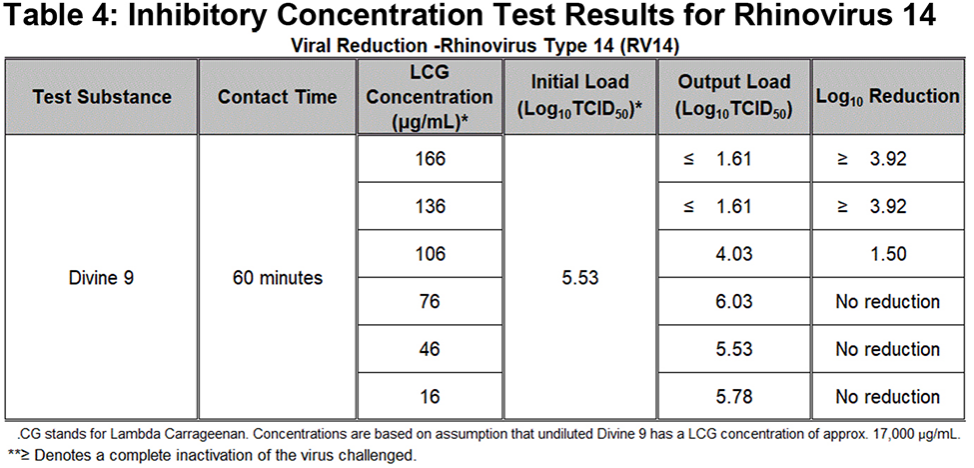
Inhibitory Concentration Test Results for Rhinovirus 14

**Results:**

In 60-minute suspension assays, complete inactivation of six common respiratory viruses of major concern to human health was observed (Table 1). A ≥4-log₁₀ reduction occurred against Influenza A (H1N1, H3N2), Avian Influenza (H5N1), SARS-CoV-2, Rhinovirus Type 14, and Respiratory Syncytial Virus (RSV) at full product concentration. An initial titration study indicated the viral inhibition threshold lies between 17 and 170 µg/mL (Table 2). In targeted concentration-response testing, both H5N1 and Rhinovirus 14 exhibited IC₅₀ values near 106 µg/mL, with ≥4-log₁₀ reductions maintained at concentrations of 136 µg/mL and above (Tables 3 and 4). No cytotoxicity or viral interference was observed in any test condition.

**Conclusion:**

A Lambda carrageenan-based formulation demonstrated high antiviral efficacy against all tested respiratory viral pathogens, including avian influenza H5N1. Strong efficacy was maintained at relatively low concentrations. The absence of cytotoxic effects and consistent performance across viral targets support its potential as a broad-spectrum antiviral agent and warrant investigation into therapeutic applications to prevent respiratory viral infections.

**Disclosures:**

James W. Arbogast, PhD, Lanvira LLC: Advisor/Consultant Cory Chiossone, MPH, Microbac Laboratories, Inc.: Employee Tanya Kapes, B.S., Microbac Laboratories, Inc.: Employee Susan Nicholson, MD FIDSA, Lanvira LLC: Advisor/Consultant

